# Construction of a Co-Expression Network for lncRNAs and mRNAs Related to Urothelial Carcinoma of the Bladder Progression

**DOI:** 10.3389/fonc.2022.835074

**Published:** 2022-02-25

**Authors:** Yeqing Mao, Chao Wen, Zitong Yang

**Affiliations:** ^1^ Urology Department, The First Affiliated Hospital, College of Medicine, Zhejiang University, Hangzhou, China; ^2^ Medical College, Zhejiang University, Hangzhou, China

**Keywords:** urothelial carcinoma of the bladder, WGCNA, COX analysis, lncRNA-mRNA co-expression network, prognostic marker

## Abstract

Carcinoma of urinary bladder is the most familiar cancer of the urinary tract, with the highest incidence in men. However, its prognosis and treatment have not improved significantly in the last 30 years. The main reason for this may be related to the alteration and regulation of genes. These alterations in genes that play a crucial role in cell cycle regulation may result in high-grade tumors and may alter drug sensitivity. Notably, the role of lncRNA in bladder cancer, especially the lncRNA-mRNA regulatory network, has not been fully elucidated. In this manuscript, we compared RNA sequencing (RNA-seq) data from 19 normal bladder tissues and 411 primary bladder tumor tissues using The Cancer Genome Atlas (TCGA) data bank, subjected differentially expressed mRNAs and lncRNAs to weighted gene co-expression network analysis, and screened out modules highly correlated with tumor progression. Subsequently, a lncRNA-mRNA co-expression network was built, and two key mRNAs were identified *via* COX regression analysis. Kaplan-Meier curve analysis revealed that the overall survival of sick people in the high-risk section was significantly shorter than those in the low-risk section. Therefore, this lncRNA-mRNA-based co-expression pattern may be used clinically to predict the prognosis of carcinoma of urinary bladder people. Our study not only provides a genetic target for carcinoma of urinary bladder therapy but also provides new ideas for people in the medical profession to discover the treatment of various tumors.

## Introduction

Carcinoma of urinary bladder is one of the most familiar malignancies worldwide (1), dominated by urothelial carcinoma of the bladder (BLCA), which accounts for approximately 90% of bladder cancers (2). It is predicted that there were 573,278 new cases of BCa and 212,536 deaths in 185 countries worldwide in 2020 (3). The traditional cure for carcinoma of urinary bladder mainly include surgical excision and chemotherapy. Hence, the recurrence rate is not low, with an overall 5-year survival rate of 15-20% (4, 5). Additionally, surgical treatment and chemotherapy are quite limited for advanced bladder cancer (6, 7). This high recurrence rate may be partly explained by the poorly understood pathogenesis of BCa (8). Therefore, exploring the pathogenesis of BCa and identifying accurate and effective biomarkers based on its clinical spectrum is vital for early diagnosis without obvious clinical symptoms, assessment of prognosis, and the development of effective treatment strategies.

For the past few years, high-throughput transcriptome sequencing has become extremely usual, revealing that up to 70% of the human genome has been transcribed (9). Hence, Most of the transcribed genes detected by high-throughput sequencing are non-coding genes, which may be associated with noncoding RNA (lncrnas) longer than 200 nucleotides (10). LncRNAs may be the most critical regulators of gene expression, cell growth, cell differentiation, cell development and chromatin dynamics (11). Thousands of lncRNAs have been shown to be mutated or aberrantly expressed in all kinds of cancers. For example, in bladder cancer, MEG3 overexpression promotes apoptosis and inhibits cell proliferation in BCa cells (12). LncRNA MALAT1, an oncogene in lung cancer, is expressed in association with metastasis and survival in lung cancer (13). However, because of our limited understanding of lncrnas, it is still a difficult task to identify lncrnas associated with cancer. One of the essential molecular mechanisms of lncRNAs is as competing endogenous RNAs (ceRNAs), which act as sponges for microRNAs (miRNAs). Aberrant expression of miRNAs has also been found in many types of cancer (14, 15). Additionally, lncRNAs may affect post-transcriptional modifications of mRNAs, suggesting that their mechanism of action is also related to the targeting of mRNAs. Thus, lncRNA-induced disruption of target-gene mRNA transcription is an effective strategy to identify cancer-critical functional lncRNAs (16, 17). Hence, because the lack of simultaneous analysis of lncRNA and mRNA expression levels in bladder cancer, few studies have reported the existence of lncRNA-mRNA regulatory networks associated with bladder cancer progression.

This study identified mRNAs and lncRNAs differentially expressed during bladder cancer progression based on 411 BLCA patient samples from The Cancer Genome Atlas (TCGA) database, subjected them to weighted gene co-expression network analysis (WGCNA), and confirmed mRNAs and lncRNAs associated with bladder cancer progression. A multi-step approach was used to construct a bladder cancer progression-associated lncRNA-mRNA co-expression network to reveal the potential roles of bladder cancer-associated mRNAs and lncRNAs. This study provides useful information for exploring potential candidate biomarkers for diagnosis, prognosis and drug targets in bladder cancer.

## Methods

### Data Source Center

RNA-Seq gene expression profiles of urothelial carcinoma of the bladder (BLCA) patients were rooted in The Cancer Genome Atlas data bank, including FPKM and count formats. Clinical information, such as survival time and survival status, was downloaded from the TCGA portal. Later, we use R software to perform data extraction and sorting, and then use the coding/non coding classification provided by gencode/Ensembl, including the classification that only produces “antisense” and “lncrna”, “lncRNA”. Subsequently, the lncRNA and mRNA expression matrices and clinical data were got.

### Differential Expression Analysis

Differential expression of mRNA and lncRNA was studied using the edgeR package of R software. Adjusted P-values were analyzed in TCGA to correct for false-positive results. “P-value < 0.05 and |logFC| ≥ 1” was defined as the threshold for lncRNA and mRNA differential expression screens, and volcano plots were painted using the R package ggplot2.

### Weighted Gene Co-Expression Network Analysis

Using the WGCNA data bank of R software, Pearson correlation coefficients between genes were got from differentially expressed mRNAs and lncRNAs used for WGCNA; later, the appropriate soft threshold β was chosen to ensure no network scalability. A one-step method was used to construct a gene network, transforming the adjacency matrix into a topological overlap matrix (TOM) and generating a hierarchical clustering tree of genes using hierarchical clustering. The DynamicTreeCut method was used to identify highly correlated co-expressed gene modules, with the threshold set to cutHeight = 0.99 and minSize = 20. The Pearson correlation test analyzed the relationship between module eigengene (ME) and clinical features.

### Functional and Pathway Enrichment Analyses

Kyoto Encyclopedia of Genes and Genomes (KEGG) and Gene ontology (GO) pathway enrichment analyses were figured out that for all genes in the blue module using Database for Annotation, Visualisation and Integrated Discovery (v6.8).

### Protein-Protein Interaction Network Construction (PPI) and Hub Gene Screening

The STRING data bank was used to identify known and predicted protein-protein interactions. STRING was also used to analyze all genes in the blue module, construct the PPI network and apply MCODE in the Cytoscape (v3.8.2) software to screen the hub genes in the PPI network.

### lncRNA-mRNA co-Expression Network Establishment

In order to clarify co-expressed lncRNA-mRNA pairs, we calculated Pearson correlation coefficients from the expression values between mRNA pairs and each differentially expressed lncRNA. The threshold of the Pearson correlation coefficient was set to > 0.5, and the corresponding FDR was set to < 0.01. In total, 248 lncRNAs and 1,308 mRNAs were identified from 57,634 co-expression relationships.

### Survival Analysis

The optimal cut-off point for risk stratification was determined using X-tile (version 3.6.1; Yale University, New Haven, CT, USA). Gene expression in BLCA was divided into high and low expression groups according to the optimal cut-off value. Kaplan-Meier survival analysis was performed on both parts, and the log-rank test difference was statistically significant (P < 0.05). All analyses were performed using R 3.1.0. Multivariate and univariate COX regression analyses were used to assess the relationship between survival and gene expression levels.

### Gene Set Enrichment Analysis

Usually, we use GSEA to divide the sample into two plates (low expression and high expression), so that we can determine the effect of gene expression on tumor. The screening conditions were FDR < 0.25 and P < 0.05.

### Cell Culture

People bladder carcinoma cell line HT-1376 and human bladder epithelial cell line HCV-29 were obtained from Cell Bank of Type Culture Collection, Chinese Academy of Sciences. The cells were cultured in RPMI-1640 medium (Invitrogen, California, USA), then added with 10% fetal bovine serum (FBS, GIBCO, California, USA), and cultured in humidified air including 5% CO_2_ at 37°C.

### qRT-PCR

Total RNA was rooted in cells using TRIzol reagent (Invitrogen, CA, USA). RNA was reverse-transcribed to cDNA by PrimeScript RT Master Mix (Takara, Japan). RT-PCR analyses were performed using the 7500 Fast Real-Time PCR System (Applied Biosystems, Forster City, CA,USA) with the primers in **Table 1**. Data were explained by 2^-∆∆Ct^ method and GAPDH was the internal reference of lncRNA DEPDC-AS1, CCNB1 and CDC20.

**Table 1:** Primer sequences.

Genes	Primer sequences
lncRNA DEPDC-AS1	Forward: 5’-TGGCCCCAACTCCTGGTGACT-3’
	Reverse: 5’-CCTGTTTTAAAAATCATTGCGGAAGCTCA-3’
CCNB1	Forward: 5’-AATAAGGCGAAGATCAACATGGC-3’
	Reverse: 5’-TTTGTTACCAATGTCCCCAAGAG-3’
CDC20	Forward: 5’-GCACAGTTCGCGTTCGAGA-3’
	Reverse: 5’-CTGGATTTGCCAGGAGTTCGG-3’
GAPDH	Forward: 5’-CTGGGCTACACTGAGCACC-3’
	Reverse: 5’-AAGTGGTCGTTGAGGGCAATG-3’

## Results

### Identification of Differentially Expressed mRNAs and lncRNAs

**Figure 1:**
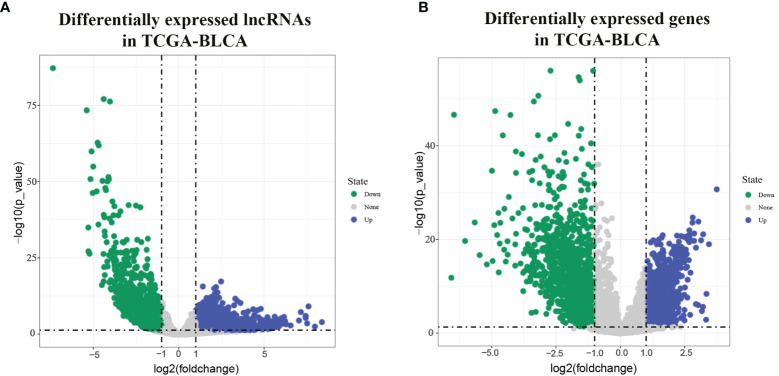
Differentially expressed lncRNAs and mRNAs in TCGA-BLCA. [**(A)** volcano diagram showing BLCA differentially expressed lncRNA in TCGA; **(B)** volcano diagram showing BLCA differentially expressed mRNA in TCGA].

In all 411 cancer tissue samples and 19 paracancer tissue samples were got from the TCGA-BLCA data bank. From the results of the screening based on lncrna expression in each sample shown by volcano plots, we can see 2168 upregulated lncrnas and 1226 downregulated lncrnas (**Figure 1A**); **Figure 1B** response screens 925 upregulated mRNAs and 1454 downregulated mRNAs.

### Identification of Gene Co-Expression Plate

**Figure 2:**
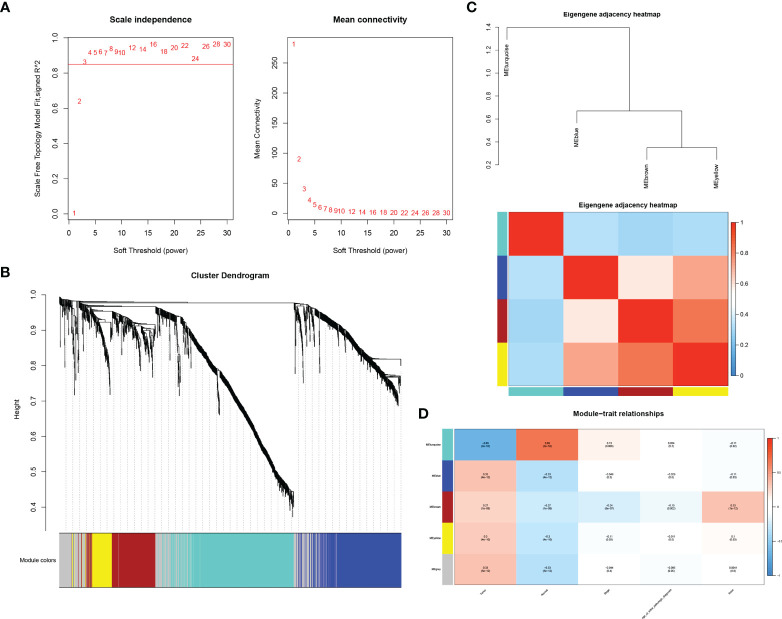
Construction of weighted gene co-expression network. [**(A)** selection of soft threshold β; **(B)** correlation heat map of neighboring modules in WGCNA; **(C)** clustering dendrogram of gene modules; **(D)** in the module feature heat map, each column corresponds to clinical parameters and each row corresponds to a module feature gene].

In order to probe the co-expression patterns of mRNAs and lncRNAs in BLCA, we screened 3,394 differentially expressed lncRNAs and 2,379 differentially expressed mRNAs obtained from the TCGA database for WGCNA analysis. A power of β=3 was set as the soft threshold of the scale-free network (**Figure 2A**). As explained in **Figure 2B**, the clustering dendrogram contained five co-expression modules, denoted by turquoise, blue, brown, yellow and grey. We explored the module functions by generating an intrinsic gene neighbor-joining heat map and found that the blue module strongly correlated with other modules. Moreover, by analyzing the correlation between each module and its clinical features, we confirmed that the blue module was a significant module highly related to BLCA tumor progression (**Figures 2C, D**).

### Enrichment Analysis of Genes in the Blue Plate

**Figure 3:**
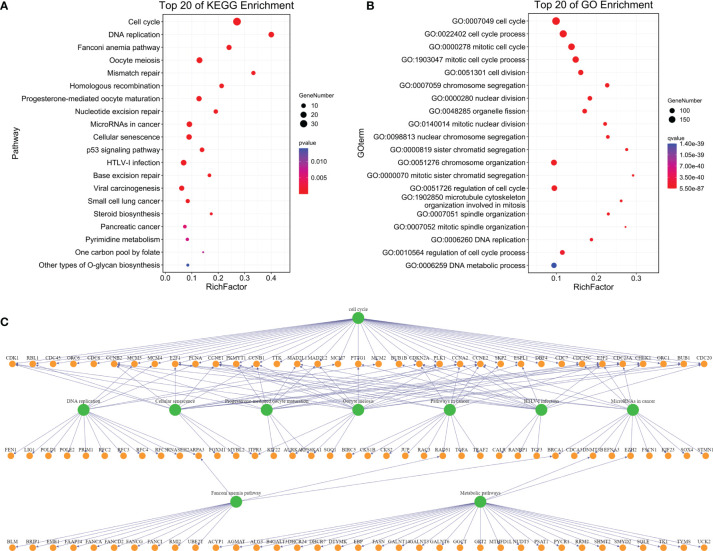
Enrichment analysis of genes in the blue module. [**(A)** Top 20 enrichment of KEGG pathway of the mRNA in a blue module; **(B)** Top 20 enrichment of GO pathway of the mRNA in the blue module; **(C)** Interaction and overlapping (of the top 10 pathways)].

In order to know the biological functions related to tumor progression in the blue plate, the co-expressed mRNAs were annotated using KEGG and GO. KEGG analysis showed that the mRNAs associated with tumor progression were significantly enriched in the cell cycle and DNA repair (**Figure 3A**); GO analysis revealed that the mRNAs associated with tumor progression were significantly enriched in the cell cycle and cell cycle progression (**Figure 3B**). The cell cycle is considered the most central function among the KEGG- and GO-enriched pathways because exchanges with other pathways strongly depend on its presence (**Figure 3C**).

### Construction of lncRNA-mRNA Co-Expression Network and PPI Network

**Figure 4:**
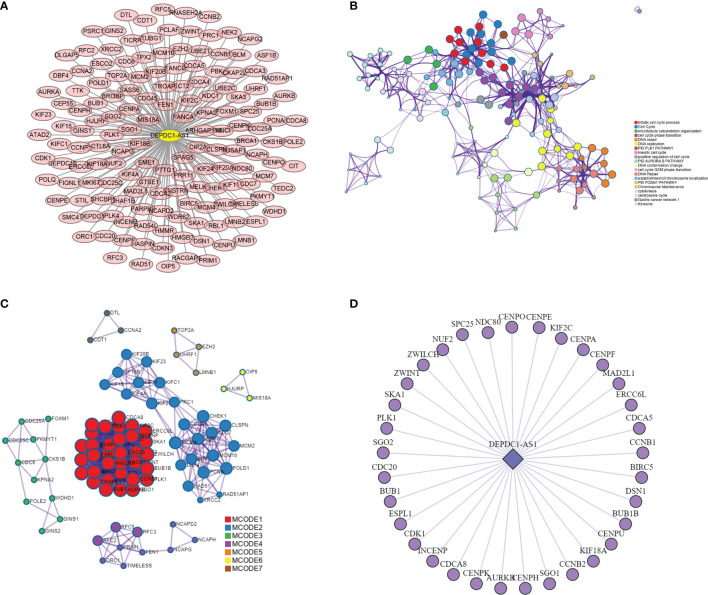
lncRNA-mRNA co-expression network and PPI network construction. [**(A)** lncRNA-mRNA co-expression network in the blue module; **(B)** protein-protein interaction network of co-expressed mRNAs in co-expression network; **(C)** MCODE plug-in screening out the highest-scoring sub-network; **(D)** co-expression network of lncRNA DEPDC1-AS1 and mRNAs in MCODE1].

By analyzing the lncRNA-mRNA co-expression patterns in the blue plate, 1,558 co-expression relationships, including those between 22 lncRNAs and 158 mRNAs, were obtained. The key lncRNA DEPDC1-AS1 and 152 co-expressed mRNAs were obtained by screening based on degree (**Figure 4A**). The 152 mRNAs were analyzed using the online analysis website STRING to obtain PPI protein network interactions and further visualize the gene information and network construction (**Figure 4B**). The properties of each node in the network graph were identified and visualized using the MCODE plugin in Cytoscape (**Figure 4C**) and the Top 3 MCODE; the functional enrichment is shown in **Table 2**. Thirty-three mRNAs in the largest sub-network were selected as key mRNAs. The co-expression network of lncRNA DEPDC1-AS1 and 33 hub mRNAs is shown in **Figure 4D**.

**Table 2:** Pathway and process enrichment analysis on Top 3 MCODE.

MCODE	GO	Description	Log_10_(P)
MCODE_1	R-HSA-2500257	Resolution of Sister Chromatid Cohesion	-75.5
MCODE_1	R-HSA-68882	Mitotic Anaphase	-69.5
MCODE_1	R-HSA-2555396	Mitotic Metaphase and Anaphase	-69.5
MCODE_2	GO:1903047	mitotic cell cycle process	-25.7
MCODE_2	R-HSA-176187	Activation of ATR in response to replication stress	-20.3
MCODE_2	GO:0033260	Nuclear DNA replication	-19.8
MCODE_3	R-HSA-69278	Cell Cycle, Mitotic	-13.6
MCODE_3	R-HSA-1640170	Cell Cycle	-12.8
MCODE_3	R-HSA-69242	S Phase	-10.8

### Prognostic Analysis of 33 Co-Expressed mRNAs in Bladder Cancer

The 33 co-expressed mRNAs were subjected to univariate and multivariate analyses (**Table 3**). The consequences of the univariate COX analysis showed that none of the 33 mRNAs was statistically significant. However, in the univariate analysis, due to the correlation between the independent variables, the effect of the independent variable on the dependent variable reflected not only its effect but also a comprehensive result after including the effect of the variable itself and the confounding effect of other variables. In the multivariate analysis, the regression plate was constructed to adjust for the effects of other confounding factors so that the factor’s actual effect on the dependent variable was revealed. Therefore, the results of the multifactorial analysis show that cell cycle protein B1 (CCNB1) and cell division cycle 20 (CDC20) are risk factors for the prognosis of carcinoma of urinary bladder patients.

**Table 3:** Univariate and multivariate Cox regression analysis of the 33 co-expressed mRNAs in BLCA patients.

Variable	Univariate analysis			Multivariate analysis		
	β	P-value	Exp (95% CI for exp)	β	P-value	Exp (95% CI for exp)
CENPU	0.077	0.355	1.080 (0.918-1.271)	0.146	0.326	1.157 (0.865-1.549)
CCNB1	0.113	0.168	1.120 (0.953-1.316)	0.509	0.026	1.663 (1.063-2.602)
KIF18A	-0.004	0.957	0.996 (0.853-1.162)	-0.445	0.029	0.641 (0.429-0.956)
BUB1B	0.121	0.145	1.128 (0.959-1.327)	0.539	0.040	1.714 (1.025-2.868)
ERCC6L	-0.083	0.314	0.921 (0.784-1.081)	-0.328	0.013	0.720 (0.555-0.934)
CDC20	0.111	0.123	1.117 (0.970-1.287)	0.420	0.046	1.522 (1.007-2.300)
BUB1	-0.001	0.988	0.999 (0.855-1.167)	-0.361	0.110	0.697 (0.448-1.086)
CCNB2	0.049	0.537	1.051 (0.898-1.229)	-0.332	0.186	0.718 (0.439-1.174)
AURKB	0.005	0.946	1.005 (0.866-1.166)	-0.222	0.238	0.801 (0.554-1.158)
BIRC5	0.087	0.273	1.090 (0.934-1.273)	0.159	0.479	1.172 (0.755-1.820)
CDCA8	0.058	0.462	1.060 (0.908-1.237)	-0.034	0.889	0.967 (0.601-1.554)
CENPA	0.057	0.433	1.059 (0.918-1.222)	0.152	0.509	1.164 (0.742-1.828)
CENPE	0.042	0.616	1.043 (0.885-1.229)	-0.171	0.439	0.843 (0.547-1.299)
CDK1	0.015	0.858	1.015 (0.860-1.198)	-0.174	0.423	0.841 (0.550-1.286)
CDCA5	0.071	0.359	1.074 (0.922-1.250)	-0.140	0.546	0.870 (0.552-1.369)
CENPF	0.054	0.468	1.055 (0.913-1.219)	0.030	0.887	1.030 (0.684-1.552)
CENPO	0.050	0.653	1.051 (0.845-1.307)	-0.085	0.716	0.918 (0.580-1.453)
CENPK	0.005	0.955	1.005 (0.846-1.194)	-0.290	0.100	0.748 (0.530-1.057)
CENPH	0.015	0.879	1.015 (0.837-1.232)	-0.020	0.908	0.980 (0.697-1.377)
DSN1	0.060	0.590	1.061 (0.855-1.318)	0.033	0.864	1.033 (0.711-1.502)
KIF2C	0.063	0.440	1.065 (0.908-1.249)	-0.193	0.510	0.824 (0.465-1.463)
MAD2L1	0.027	0.744	1.027 (0.873-1.209)	0.002	0.992	1.002 (0.685-1.465)
INCENP	0.128	0.159	1.137 (0.951-1.358)	0.437	0.058	1.547 (0.986-2.429)
ESPL1	0.087	0.276	1.091 (0.933-1.277)	0.127	0.496	1.136 (0.787-1.638)
NDC80	0.029	0.696	1.030 (0.890-1.191)	0.005	0.978	1.005 (0.707-1.428)
NUF2	-0.022	0.746	0.978 (0.856-1.118)	-0.240	0.133	0.786 (0.575-1.076)
SGO2	0.077	0.432	1.080 (0.891-1.309)	0.078	0.758	1.082 (0.657-1.781)
SGO1	-0.044	0.570	0.957 (0.821-1.115)	-0.189	0.292	0.828 (0.582-1.177)
PLK1	0.099	0.177	1.104 (0.956-1.276)	0.156	0.370	1.169 (0.831-1.644)
SKA1	0.097	0.232	1.102 (0.940-1.292)	0.392	0.052	1.480 (0.996-2.199)
SPC25	0.014	0.860	1.015 (0.864-1.191)	-0.103	0.623	0.902 (0.598-1.360)
ZWINT	0.045	0.583	1.046 (0.890-1.230)	0.016	0.933	1.016 (0.702-1.470)
ZWILCH	0.083	0.493	1.086 (0.857-1.377)	0.187	0.415	1.205 (0.770-1.886)

### Prognostic Analysis of lncRNA DEPDC-AS1, CCNB1 and CDC20 in BLCA

**Figure 5:**
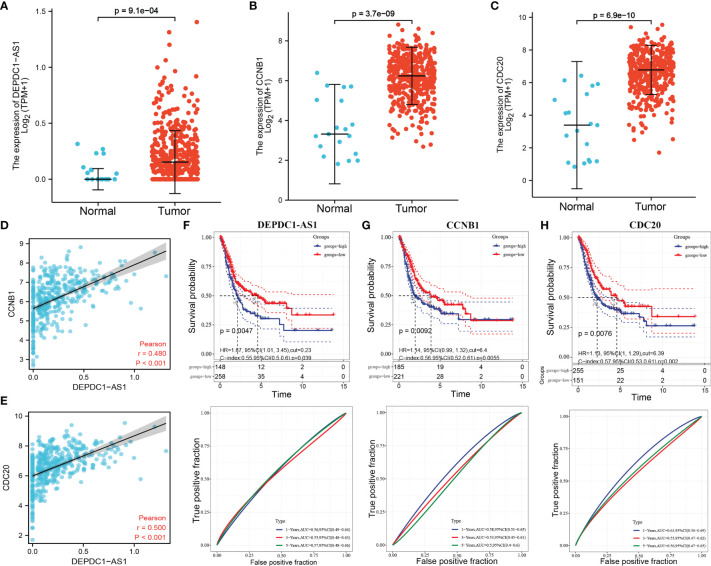
Expression and prognostic analysis of lncRNA and mRNA in BLCA. [**(A)** DEPDC1-AS1 expression in BLCA; **(B)** CCNB1 expression in BLCA; **(C)** CDC20 expression in BLCA; **(D)** correlation between DEPDC1-AS1 and CCNB1 expression in BLCA; **(E)** correlation between DEPDC1-AS1 and CDC20 expression in BLCA; **(F)** KM curve of DEPDC1- AS1 expression with KM curve and time-dependent ROC curve of overall survival; **(G)** KM curve and time-dependent ROC curve of CCNB1 expression with overall survival; **(H)** KM curve and time-dependent ROC curve of CCNB1 expression with overall survival].

As shown in **Figures 5A**–**C**, lncRNA DEPDC-AS1 as well as CCNB1 and CDC20 had significantly higher expressions in carcinoma of urinary bladder tissues compared to normal paracancerous tissues. Additionally, the expression of DEPDC-AS1 was significantly and positively correlated with the expression of CCNB1 and CDC20 in BLCA (**Figures 5D, E**). DEPDC-AS1, CCNB1 and CDC20 expressions were classified into low and high expression groups according to the optimal cut-off values; the survival analysis results revealed that the high expression of DEPDC-AS1, CCNB1 and CDC20 was significantly related to poor prognosis in BLCA patients, ROC results showed that the AUC for 1 year of DEPDC-AS1, CCNB1 and CDC20 was 0.56, 0.58 and 0.63, 3 years of DEPDC-AS1, CCNB1 and CDC20 was 0.55, 0.53 and 0.65, of DEPDC-AS1, CCNB1 and CDC20 was 0.57, 0.55 and 0.56 (**Figures 5F**–**H**). Despite the results of AUC value was not good enough, but it might be of some value when in combination.

### Gene Set Enrichment Analysis of CDC20 and CCNB1

**Figure 6:**
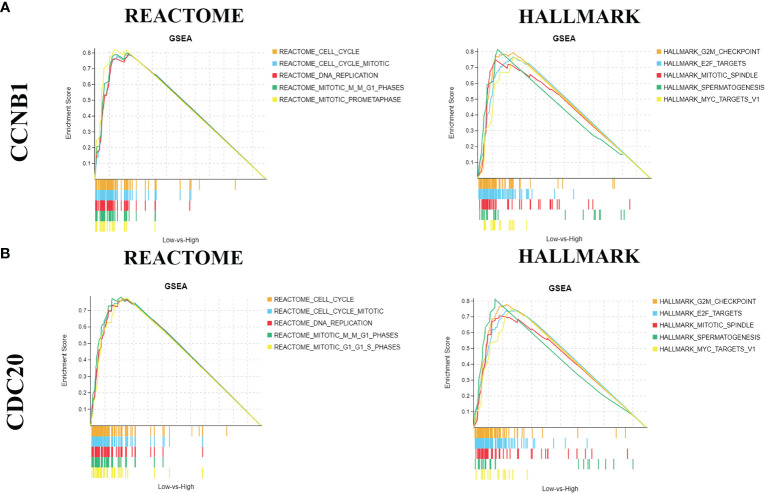
Gene set enrichment analysis. [**(A)** REACTOME and HALLMARK pathway analysis of CCNB1; **(B)** BREACTOME and HALLMARK pathway analysis of CDC20].

We wanted to find out all the functions of ccnb1 and Cdc20, using GSEA and classifying the top 5 enriched pathways (FDR Q-value < 0.250, NOM p-value < 0.050) based on the normalized enrichment score (NES). As shown in **Figure 6**, the Reactome Database enrichment results showed that both CCNB1 and CDC20 were mainly enriched in cell-cycle and DNA-replication pathways. The Hallmark enrichment results showed that both CCNB1 and CDC20 were positive for G2M checkpoint and E2F targets.

### The Expression of CCNB1, lncRNA DEPDC-AS1 and CDC20 in BC Cells

**Figure 7:**
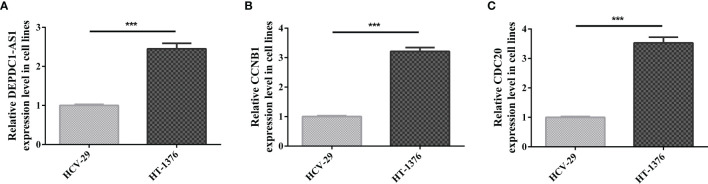
Relative expression of genes quantified by qRT-PCR. [**(A)** Relative expression level of lncRNA DEPDC-AS1; **(B)** Relative expression level of CCNB1; **(C)** Relative expression level of CDC20; ***P < 0.001].

The expression of lncRNA DEPDC-AS1, CCNB1 and CDC20 in carcinoma of urinary bladder cell line was determined by qRT-PCR analysis. The results figured out that the expression of lncRNA DEPDC-AS1, CCNB1 and CDC20 were significantly increased in HT-1376 cells compared with that in HCV-29 (**Figures 7A**–**C**). *In vitro* experiments results reflected the same results of the biology information analysis.

## Discussion

Lncrnas are emerging as regulators of a wide range of biological functions. These newly characterized regulators play important and broad roles in cancer development and progression (18). However, the biological functions of most lncRNAs involved in epigenetic regulation and their role in risk stratification and prognosis have not been investigated. To date, some lncRNAs, such as UCA1, HHOTAIR and H19, have been detected in BCa cells. LncRNA overexpression can promote chemoresistance by regulating the Wnt signalling pathway and may serve as a potential diagnostic biomarker for BCa (19). However, there is a lack of comprehensive database providing resources for experimental validation of lncRNA functions. Experimental validation of the roles of the numerous lncRNAs is complex, laborious and very expensive. Biology information analysis is an approach increasingly used for target gene and protein studies. WGCNA is a systems biology approach for characterizing correlation patterns among genes in microarray samples, allowing the identification of modules of highly correlated genes for the study of potential functions (20). Recent researches have shown that WGCNA has been widely used for the screening and identification of disease susceptibility genes and candidate targets (21, 22). Another method is that we can also integrate the expression profiles of protein coding genes and lncrna into the co-expression model, so as to study the characteristics of lncrna in different biological processes and cancers.

Numerous researched have figured out that the occurrence of BCa is highly related to the abnormal expression of non-coding RNAs and protein-coding genes. Wang et al. combined the miRNA mRNA regulatory network, lncrna miRNA regulatory network and lncrna mRNA co expression network to obtain a three-layer network, and then calculated the topological characteristics of each node in the network, including degree, compactness and intermediation. They also identified mirna-93 and mirna-195 as controllers of three-layer networks related to BCA and regulators of many target genes, the imbalance of these target genes may be closely related to the pathogenesis of BCa (23). However, few studies have reported the existence of a lncRNA-mRNA co-expression network associated with BCa progression. LncRNAs are more likely to be co-expressed with neighboring coding genes through a cis-regulatory mechanism (24). Previous studies have shown that some lncRNAs can be co-expressed with the corresponding coding genes based on the ceRNA theory (17). Based on this theory, we analyzed and characterized the lncRNA-mRNA co-expression network in bladder cancer.

This manuscript used differentially expressed mRNAs and lncRNAs in TCGA-BLCA for WGCNA to build a functional lncRNA-mRNA co-expression network related to carcinoma of urinary bladder progression. A key lncRNA (DEPDC1-AS1) and two mRNAs (CCNB1 & CDC20) were then identified *via* univariate and multivariate COX analyses. Studies targeting DEPDC1-AS1 alone in cancer have not been reported. However, LuLu et al. (25) first demonstrated that DEPDC1-AS1 was associated with the prognosis of lung adenocarcinoma, and Yuan C et al. (26) constructed a new triple-length non-coding RNA risk scoring system for predicting the prognosis of triple-negative breast cancer, which included DEPDC1-AS1. While the present study demonstrated for the first time that DEPDC1-AS1 was associated with the prognosis of BLCA, its relevant mechanism of action in BLCA needs to be further explored. CCNB1 is a monitoring protein that initiates the process from the G2 phase to mitosis. Numerous studies have reported that CCNB1 is overexpressed in many tumors—hepatocellular carcinoma (27), colon cancer (28), and pancreatic cancer (29)—and promotes tumor cell proliferation. A recent study identified CCNB1 as a potential target and a new key biomarker for the prognosis of human BCa (30); this study’s results confirm this. In cell cycle progression, CDC20 is an important control factor of cell cycle checkpoints. Its most important function is to bind to the late promoter complex (APC/C), which in turn regulates the degradation of the isolate inhibitor protein (31, 32). Thus, dysregulation of CDC20 may have a considerable impact on cell growth and tumorigenesis. Furthermore, many studies have figured out that CDC20 is a carcinogen that promotes cancer development (33, 34). Choi et al. (35) found elevated CDC20 expression in patients with uroepithelial bladder cancer (UBC) and that high CDC20 expression in UBC patients was related to short recurrence-free survival and poor overall survival. The results of this study confirmed these findings. Additionally, the gene set enrichment analysis results showed that the CCNB1 and CDC20 enrichment pathways are similar in that both act on the regulatory mechanisms of BCa development by regulating the progression of the cell cycle.

In addition, lncRNA can play multiple roles in the regulation of its target genes. Firstly, lncRNA can play a role through cis or trans regulation of its target genes (36, 37). A single lncRNA may regulate multiple target genes through different mechanisms. Secondly, lncRNA can also be used as a guide to promote DNA protein interaction. It should be noted that lncRNA can play a role as enhancer RNA as well when recruiting protein complexes to induce DNA cyclization and target gene transcriptional activation (38). Thirdly, lncRNAs can also be used as bait to bind to proteins involved in transcription to prevent them from binding to DNA target proteins, or as competitive endogenous RNA (ceRNAs) to bind to miRNAs to prevent negative regulation of target genes (39). The binding of lncRNA to mRNA also leads to intron retention, which promotes the selective splicing of mRNAs (40). Chen et al. (41), found that LncRNA PVT1 accelerates malignant phenotypes of bladder cancer cells by modulating miR-194-5p/BCLAF1 axis as a ceRNA. However, there were no reports on the mechanism of how DEPDC1-AS1 works in the regulation of the cancer progression. Luckily, this provide us a novel insights into and a new research direction of the DEPDC1-AS1 in cancers.

In conclusion, our analysis by synthesis provided new insights into the lncRNA-mRNA co-expression network in bladder cancer progression. It indicates that lncRNA plays an important role in bladder cancer. LncRNA-DEPDC1-AS1 may serve as a biomarker to predict survival in BCa patients. Moreover, it may mediate the BCa cell cycle through a co-expression network involving CCNB1 and CDC20, thus affecting BCa progression. In addition, the biological function of lncRNA-DEPDC1-AS1 requires further laboratory characterization and study. Therefore, the results and conclusions obtained in this study may provide an essential theoretical basis for future experimental studies on lncRNA’s role in bladder cancer.

## Data Availability Statement

The original contributions presented in the study are included in the article/supplementary material. Further inquiries can be directed to the corresponding author.

## Ethics Statement

Ethical approval/written informed consent was not required for the study of animals/human participants in accordance with the local legislation and institutional requirements.

## Author Contributions

All authors listed have made a substantial, direct, and intellectual contribution to the work, and approved it for publication.

## Conflict of Interest

The authors declare that the research was conducted in the absence of any commercial or financial relationships that could be construed as a potential conflict of interest.

## Publisher’s Note

All claims expressed in this article are solely those of the authors and do not necessarily represent those of their affiliated organizations, or those of the publisher, the editors and the reviewers. Any product that may be evaluated in this article, or claim that may be made by its manufacturer, is not guaranteed or endorsed by the publisher.
